# Determinants of Childhood Asthma: A Case Control Study from a Tertiary Care Hospital in Bengaluru, South India

**DOI:** 10.34763/jmotherandchild.20232701.d-22-00059

**Published:** 2023-08-31

**Authors:** Andrea Daniella Johnson, Alex Fernando, Maria Lewin, Farah Naaz Fathima

**Affiliations:** St. John's Medical College, Bangalore, Karnataka, India; Department of Paediatrics, St. John's Medical College, Bangalore, Karnataka, India; Department of Community Health, St. John's Medical College, Bangalore, Karnataka, India

**Keywords:** childhood, asthma, determinants, risk factors

## Abstract

**Background:**

Childhood asthma is a common, and often serious, chronic disease with episodic exacerbations in infants and children. There is an increasing trend in the prevalence of childhood asthma in developing countries. Objectives: To identify the determinants of childhood asthma.

**Methods:**

A case control study with 30 cases of childhood asthma and 30 gender- and aged-matched controls selected from the paediatric outpatient department and paediatric ward of a tertiary hospital. The primary caregiver was interviewed to capture sociodemographic details, prenatal and birth history, and history of exposure to environmental risk factors. Odds ratios with 95% confidence intervals were calculated to determine the strength of association between childhood asthma and independent co-variates, followed by subgroup multiple logistic regression analysis.

**Results:**

We found that children with a parental history of allergy/atopy [OR=2.88 (1.94–4.27), P<0.001], residence in houses located in industrial areas [AOR=2.72 (2.6–323.1), P<0.001], exposure to incense at home [AOR=2.03 (1.14–29.42), P<0.001], or a history of allergic rhinitis [AOR=3.09 (2.22–243.25), P<0.001] had significantly higher odds of developing childhood asthma.

**Conclusion:**

Our study found that having homes located in industrial areas, burning incense at home, parental history of allergy, and history of allergic rhinitis in the child are determinants of childhood asthma. The findings from our study can be used to generate awareness regarding risk factors that are linked to childhood asthma.

## Introduction

Asthma is a chronic disease with episodic exacerbations involving inflammation of the bronchial airways, which results in symptoms such as shortness of breath, coughing, wheezing or exercise intolerance [1]. Childhood asthma is a common, and often serious, chronic disease in infants and children. The prevalence of childhood asthma is increasing among low-and middle-income countries [2]. ISAAC (International Study of Asthma and Allergies in Children) conducted surveys in 97 countries between 2000 and 2003 and found that about 14% of the world's children were likely to have asthmatic symptoms [3]. Recent studies on childhood asthma conducted across India have shown its prevalence to be ranging from 7.9% to 13.1% in school-going children [4, 5]. A study conducted in Bangalore by Paramesh, who studied 20,000 children under the age of 18 years, has shown that the prevalence of asthma has increased over threefold over two decades, from 9% in 1979 to 29.5% in the year 1999 [6]. It has been reported in the United States that asthma among school-going children was responsible for 7 million missed school days and an annual cost of $211 million. [7].

Several risk factors are known to be associated with asthma, both intrinsic or extrinsic, which result in the varying severity of asthma. These include familial history, birth weight, breastfeeding, environment, exercise, furry pet ownership, smoking habits among family members, absence of smoke outlet in the house, and other allergies like dermatitis and/or rhinitis [8,9,10,11].

There is a dearth of studies that focus on risk factors for childhood asthma in the Indian subcontinent. Our study aims to identify the determinants of childhood asthma. The results of this study will help to generate evidence to develop and design targeted interventions to raise awareness to prevent or control these risk factors.

## Methods

*Study design and setting:* This was a case control study conducted at a tertiary care teaching hospital in Bengaluru from 2017 to 2019.

*Study Population:* We studied children aged from two years to less than 18 years attending the paediatric outpatient department or who had been admitted to the paediatric inpatient wards.

*Sample size estimation:* Sample size was calculated online [12] with reference to a previous study by Majeed et al. [9] which reported that 16% of cases and 4.5% of controls had a family history of allergic rhinitis with odds ratio of 4. With a power of 80%, alpha error of 5%, and a 1:1 ratio of cases to controls, we estimated a requirement of 30 cases (children with paediatric asthma) and 30 controls (children without paediatric asthma).

*Selection of cases and controls:* Cases were children diagnosed with childhood asthma based on physician assessment using the Global Initiative for Asthma (GINA) guidelines. Controls were children who did not have childhood asthma, nor any symptoms suggestive of asthma, and were chosen from the same study setting. For each case that was selected, we selected a control matched for gender and age (± 3 months). Cases and controls were consecutively sampled until the sample size was achieved.

*Exclusion criteria:* Seriously ill children admitted to the paediatric intensive care unit of the hospital and children with congenital malformations or chronic systemic diseases were excluded from the study.

*Assessment of exposure:* An extensive review of literature was completed in order to prepare an interview schedule which was face-validated by two experts in the field of paediatrics and pulmonary medicine. The study tool was administered to the primary caregiver of the child in the local language. The study tool contained questions pertaining to sociodemographic details of the child and family, as well as possible risk factors associated with childhood asthma such as prenatal and birth history of the child, feeding practices, details of illnesses and treatment during the first year of life, and family history of asthma/allergies. We also extensively explored housing and environmental conditions that could predispose to childhood asthma like overcrowding or pets in the house, type of cooking fuel, exposure to tobacco smoke and other indoor air pollutants, material of the child's pillow cover, use of carpets/rugs, frequency of washing the child's bed linen, child's usage of soft toys, potentially industrial location of family home, and family home's distance from main road.

*Operational definitions*: ‘Experienced respiratory problems in the neonatal period’: fast, noisy, or difficult breathing in the first four weeks of life. ‘Any illness in the first year’: any condition for which the infant was taken to a healthcare facility or for which the advice of healthcare personnel was sought. Socioeconomic status was determined using the Modified BG Prasad classification [13]. Anthropometry (weight and height) of the child was documented using standard tools and procedures, and was interpreted using the World Health Organization (WHO) growth charts (weight for height and BMI for age).

*Ethical Considerations:* An Institutional Ethics Clearance for the study was obtained prior to commencement of the study (IEC#45/2017). Written informed consent from the parent was taken. Verbal assent from children over the age of seven was obtained for anthropometric assessment.

*Statistical Analysis*: The data was entered in Microsoft Excel and analysed using the IBM Statistical Package for Social Sciences (SPSS v17). Study variables were described using frequencies and proportion. The strength of association between childhood asthma and independent covariates was evaluated by calculating odds ratios with 95% confidence intervals. Multivariate logistic regression and sub-group analysis was completed to adjust for confounding variables, with cases and controls as the dependent variable and sociodemographic, neonatal, infant, and environmental factors as independent covariates. A penalized odd ratio (Firthlogit regression) was completed to account for the presence of 0 value in any of the cells. A *P*-value of <0.05 was considered significant for all analyses.

## Results

A total of 60 children were included in our study. 30 were cases with childhood asthma and 30 were age- and gender-matched controls. Among the study subjects, 55% were under the age of five, 60% were male, and 51% belonged to the upper or upper-middle socioeconomic group. The sociodemographic details of the study sample are described in Table 1.

**Table 1.** Sociodemographic factors in childhood asthma N=60.

**Variable**	**Category**	**Cases (30)** **N (%)**	**Controls (30)** **N (%)**	**Odds Ratio** **(95% CI)**	***P*-value**
Age	Under 5	17 (56.7)	16 (53.3)	1.14 (0.41–3.16)	0.79
	Above 5yrs	13 (43.3)	14 (46.7)		
Gender	Male	18 (60.0)	18 (60.0)	1.00 (0.36–2.81)	0.99
	Female	12 (40.0)	12 (40.0)		
Maternal education	Up to High school	20 (66.7)	25 (83.3)	0.4 (0.19–1.36)	0.14
	Graduate/Postgraduate	10 (33.3)	5 (16.7)		
Socioeconomic status	Upper/upper-middle	18 (60.0)	13 (43.3)	1.96 (0.70–5.48)	0.19
	Middle/Lower-middle/lower	12 (40.0)	17 (56.7)		

Over 61.7% of the participants were firstborn children. Most of the subjects (80%) were born full term (more than 37 completed weeks of gestation), 70% were born via vaginal delivery, and 30% had a birth weight less than 2500g. Around a quarter (23.3%) of the parents of children had a history of allergy/atopy. Risk factors during the antenatal period and birth are depicted in Table 2.

**Table 2.** Pre-natal, genetic and birth factors in childhood asthma N=60.

**Variable**	**Category**	**Cases (30)** **N (%)**	**Controls (30)** **N (%)**	**Odds Ratio** **(95% CI)**	***P*-value**
Parity index of mother	Primipara	9 (30.0)	9 (30.0)	1.0 (0.33–3.01)	0.99
	Multipara	21 (70.0)	21 (70.0)		
Birth order	1	16 (53.3)	21 (70.0)	0.49 (0.17–1.41)	0.18
	≥2	14 (46.7)	9 (30.0)		
Parental history of allergy / atopy	Yes	14 (46.7)	0 (0)	3.98 (1.10–6.86)	0.007^*^
	No	16 (53.3)	30 (100)		
Gestational age at birth	Preterm	5 (16.7)	7 (23.3)	0.66 (0.18–2.36)	0.52
	Full term	25 (83.3)	23 (76.7)		
Mode of delivery	Vaginal (normal/assisted)	19 (63.3)	23 (76.7)	0.53 (0.17–1.62)	0.26
	Caesarean section	11 (36.7)	7 (23.3)		
Birth weight	< 2500 g	4 (13.3)	8 (26.7)	0.42 (0.11–1.59)	0.17
	≥ 2500 g	26 (86.7)	22 (73.3)		

* Penalized odd ratio (Firthlogit regression) to account for 0 value in one of the cells.

Two-thirds (68.3%) of the children were exclusively breastfed for 6 months, whereas pre-lacteal feeds were given to 40.0% of the children. It was found that 5% of the children were underweight and 18% were overweight. One fourth (25.0%) experienced respiratory problems in the neonatal period and 28.3% were admitted to the neonatal intensive care unit [NICU]. Around 60% experienced illness in the first year of life, 41.7% had a history of allergic rhinitis, and 10.0% had a history of skin allergy. More than two-thirds (70.0%) had normal nutritional status. Risk factors in the neonatal period, infancy, and childhood are depicted in Table 3.

**Table 3.** Risk factors for childhood asthma in neonatal period, infancy and childhood N=60.

**Variable**	**Category**	**Cases (30)** **N (%)**	**Controls (30)** **N (%)**	**Odds Ratio** **(95% CI)**	***P*-value**
Received pre-lacteal feeds	Yes	13 (43.3)	11 (36.7)	1.32 (0.47–3.72)	0.60
	No	17 (56.7)	19 (63.3)		
Exclusive breastfeeding for 6 months	Yes	17 (56.7)	24 (80.0)	0.33 (0.1–1.03)	0.052
	No	13 (43.3)	6 (20.0)		
Experienced respiratory problems in the neonatal period	Yes	12 (40.0)	3 (10.0)	6.00 (1.48–24.3)	**0.01**
	No	18 (60.0)	27 (90.0)		
NICU admission	Yes	11(36.7)	6 (20.0)	2.32 (0.72–7.41)	0.15
	No	19(63.3)	24 (80.0)		
Any illness in the first year	Yes	24 (80.0)	12 (40.0)	6.00 (1.89–19.04)	**0.002**
	No	6 (20.0)	18 (60.0)		
Child experiences symptoms of allergic rhinitis	Yes	23(76.7)	2 (6.7)	46 (8.7–243.25)	**<0.001**
	No	7 (23.3)	28 (93.3)		
History of atopy/allergy	Yes	6 (20.0)	0 (0)	2.78 (0.14–5.71)	0.06^*^
	No	24 (80.0)	30 (100)		
Nutritional status (present)	Overweight	5 (16.7)	9 (30.0)	-	-
	Normal	22 (73.3)	20 (66.7)	0.33 (0.03–3.21)	0.34
	Underweight	3 (10.0)	2 (3.3)	0.91 (0.49–1.67)	0.76

* Penalized odd ratio (Firthlogit regression) to account for 0 value in one of the cell.

The majority (61.7%) of the study participants lived in an urban area, 23.3% lived in an industrial area, and 38.3% lived in a house near the main road. Nearly half (46.7%) of the houses were overcrowded. The proportion of children exposed to smoke, firewood, incense, and mosquito repellents at home were 18.3%, 15.0%, 40.0% and 38.3% respectively. Around half (43.3%) of the children played with soft toys. Pets were present in 31.7% of households. Around one third (35.0%) had heavy carpets/curtains at home. Risk factors for childhood asthma in the environment and within the house are depicted in Table 4.

**Table 4.** Environment and housing factors in childhood asthma N=60.

**Variable**	**Category**	**Cases (30)** **N (%)**	**Controls (30)** **N (%)**	**Odds Ratio** **(95% CI)**	***P*-value**
Residence	Rural	12 (40.0)	19 (63.3)	0.87 (0.31–2.46)	0.79
	Urban	18 (60.0)	11 (36.7)		
Located in industrial area	Yes	13 (13.3)	1 (3.3)	22.18 (2.66–184.79)	**0.001**
	No	17 (86.7)	29 (96.7)		
Location of house	Main road	15 (50.0)	8 (26.7)	2.75 (0.93–8.1)	0.06
	Away from main road	15 (50.0)	22 (73.3)		
Overcrowding	Yes	16 (53.3)	12 (40.0)	1.71 (0.62–4.77)	0.30
	No	14 (46.7)	18 (60.0)		
Exposure to tobacco smoke	Yes	6 (20.0)	5 (16.7)	1.25 (0.34–4.64)	0.74
	No	24 (80.0)	25 (83.3)		
Exposure to firewood	Yes	3 (10.0)	6 (20.0)	0.44 (0.1–1.97)	0.28
	No	27 (90.0)	24 (80.0)		
Exposure to incense	Yes	17 (56.7)	7 (23.3)	4.3 (1.41–13.07)	**0.01**
	No	13 (43.3)	23 (76.7)		
Exposure to mosquito repellants	Yes	15 (50.0)	8 (26.7)	2.75 (0.93–8.1)	0.06
	No	15 (50.0)	22 (73.3)		
Kerosene for cooking	Yes	6 (20.0)	7 (23.3)	0.82 (0.24–2.81)	0.75
	No	24 (80.0)	23 (76.7)		
Pets in the house	Yes	12 (40.0)	7 (23.3)	2.19 (0.72–6.7)	0.17
	No	18 (60.0)	23 (76.7)		
Synthetic material of child's pillow cover	Yes	4 (13.3)	8 (26.7)	0.42 (0.11–1.6)	0.2
	No	26 (86.7)	22 (73.3)		
Regular washing child's bedlinen	Yes	27 (90.0)	24 (80.0)	2.25 (0.51–9.99)	0.28
	No	3 (10.0)	6 (20.0)		
Child plays with soft toys	Yes	15 (50.0)	11 (36.7)	1.73 (0.62–4.84)	0.32
	No	15 (50.0)	19 (63.3)		
Cockroach infestation at home	Yes	22 (73.3)	17 (56.7)	2.1 (0.71–6.22)	0.18
	No	8 (26.7)	13 (43.3)		
Heavy carpet/curtain/rugs at home	Yes	15 (50)	6 (20)	4.00 (1.27–12.58)	**0.02**
	No	15 (50)	24 (80)		

Significantly higher odds of developing childhood asthma were found in children with parental history of allergy/atopy [OR=2.88 (1.94–4.27), *P*<0.001], those who experienced respiratory problems in neonatal period [OR=6.0 (1.48–24.3), *P*=0.01], those with any illness in the first year of life [OR=6.0 (1.89–19.04), *P*=0.002], children with allergic rhinitis [OR=4.6 (8.7–243.2), *P*<0.001], children with a history of skin allergy [OR=2.25 (1.65–3.03), *P*=0.02], children with houses located in industrial areas [OR=22.18 (2.66–184.8), *P*<0.001], those exposed to incense at home [OR=4.3 (1.41–13.07), *P*<0.01] and those with heavy carpets/curtains at home [OR=4.0 (1.2–12.5), *P*=0.02].

Multivariate logistic regression analysis was performed on a model which included all risk factors for childhood asthma that showed a significant odds ratio on bivariate analysis. None of the risk factors showed a significant association with childhood asthma in this model. Sub-group multiple logistic regression analysis with all environmental risk factors, adjusted for parental history of allergy, showed that children with houses located in industrial areas [AOR=2.72 (2.6–323.1), *P*<0.001] and those with history of burning incense at home [AOR=2.03 (1.14–29.42), *P*<0.001] had significantly higher odds of developing childhood asthma. Subgroup regression analysis with all risk factors in neonatal period and infancy, adjusted for parental history of allergy, showed that children with history of allergic rhinitis [AOR=3.09 (2.22–243.25), *P*<0.001], had significantly higher odds of developing childhood asthma.

## Discussion

Primary prevention of chronic disease involves the elimination or modification of risk factors for that disease [14]. In our study, we found that the risk factors for childhood asthma were having homes located in industrial areas, history of burning incense at home, parental history of allergy, and history of allergic rhinitis in the child.

In a cohort of 5654 children in Sweden, parental asthma, small-for-gestational age, and male gender were linked to childhood asthma [15]. The Nutrition Evidence Systematic Review team in the USA has published their finding based on a review of 44 studies, which shows that ever breastfeeding is protective against asthma, and that longer duration and exclusivity of breastfeeding offers even greater protection [16]. Determinants found in other studies include male gender, urban locality, low socioeconomic status, damp environment, use of firewood for cooking, presence of pets, family history of asthma/atopy, preterm birth, high body mass index, presence of a smoker at home, and lack of exclusive breastfeeding [17, 18]. A United States National Survey, which included 90,721 children, observed that preterm birth was associated with an increased risk of pre-school wheezing and school-age asthma, independent of birth weight [19]. In our study, except the for parental history of allergy, none of the above determinants were significantly associated with childhood asthma. Asthma and other allergic conditions show familial aggregation. This is due to a complex interplay of hereditary and environmental causes. A multicentric study among children from seven cities in China revealed that the odds of childhood asthma increased 16-fold among children with two asthmatic parents. This study also found that having even one grandparent with asthma doubled the risk of childhood asthma, reinforcing the significance of family history. [20] In addition, exposure to common environmental risk factors among parents and children due to shared living conditions also contributes to familial aggregation of asthma [21].

Our findings show that incense burning and location of house in an industrial area were associated with childhood asthma. A study in China also reported a significant association between incense burning and traffic-related air pollution [22]. Incense burning emits a complex mixture of particulate matter, gases, volatile organic compounds, heavy metals, and other gaseous compounds.

When inhaled, these substances elevate the oxidative stress, impair the lung defences, and lead to declined lung function [23]. The results of our study showed that children with allergic rhinitis had higher odds of developing childhood asthma. Both these conditions demonstrate similar responses at the cellular level and trigger the inflammatory cascade. They differ only in the location of the eosinophil infiltration, depending on whether it is the nasal or bronchial epithelium [24]. Health education should be given to mothers of children with allergic rhinitis regarding reducing exposure to triggers of asthma, including incense.

This hospital-based case control study among children aged two to less than 18 years studied an exhaustive list of possible risk factors. The findings from our study can be used to generate awareness regarding risk factors that are linked to childhood asthma. In clinical practice, the presence of these factors can raise the index of suspicion of childhood asthma. The findings of our study also have policy implications. Municipal authorities should consider approving locations of residential areas away from industrial zones. Children with parental history of allergy and atopy need to be monitored to diagnose childhood asthma early. More research is needed to explore the role of environmental risk factors in childhood asthma in developing countries.

*Strengths and limitations of the study:* Our study looked at an extensive list of possible risk factors for childhood asthma. So far, there has been no study in India with such an exhaustive list of determinants. Our case-control study design is robust and appropriate for identifying determinants of childhood asthma. However, the limited sample size may have contributed to a lack of significant association with traditional risk factors such as exposure to tobacco smoke, breastfeeding, and prematurity. The questions pertaining to past history and events might have resulted in a recall bias. We were also not able to quantify exposure to environmental risk factors in our study.

## Conclusion

Our study found that homes located in industrial areas, burning incense at home, parental history of allergy, and history of allergic rhinitis in the child are determinants of childhood asthma. The findings from our study can be used to generate awareness regarding risk factors that are linked to childhood asthma.

### Key messages

The study identifies determinants of childhood asthma in an Indian setting: homes located in industrial areas, history of burning incense at home, parental history of allergy, and history of allergic rhinitis in the child.
